# Supplementary material for: Do goats recognise humans cross-modally?
Source: PeerJ. 2025 Jan 14;13:e18786. doi: 10.7717/peerj.18786 (PMC11740737; doi:10.7717/peerj.18786)
Supplement: Supplemental Information 1 [file peerj-13-18786-s001.docx]

**Do goats recognise humans cross-modally? Supplementary information**

Marianne A. Mason^1,2*^, Stuart Semple^1^, Harry H. Marshall^1,3^ and Alan G. McElligott^4,5*^

*^1^ School of Life and Health Sciences, University of Roehampton, London, UK*

*^2^ Department of Animal and Veterinary Sciences, Aarhus University, Tjele, Denmark*

*^3^ RSPB Centre for Conservation Science, Cambridge, UK*

*^4^ Department of Infectious Diseases and Public Health, Jockey Club College of Veterinary Medicine and Life Sciences, City University of Hong Kong, Hong Kong SAR, China*

*^5^ Centre for Animal Health and Welfare, Jockey Club College of Veterinary Medicine and Life Sciences, City University of Hong Kong, Hong Kong SAR, China*

**Table S1:**

Subject name, year in which they were tested, sex (M = Male; F = Female), breed, age, number of years at study site, order of trials included in analysis (C = Congruent, I = Incongruent) and whether cardiac, as well as behavioural data had been successfully taken during experimental trials (Y = Yes; N = No).

| **Goat ID** | **Tested** | **Sex** | **Breed** | **Age (years)** | **Duration at Sanctuary (years)** | **Trial Sequence** | **Contributed Cardiac Data?** |
| --- | --- | --- | --- | --- | --- | --- | --- |
| Bernard  Davey  Dixie  Dylan  Ewok  Juliet  Luke  Natalie  Nigel  Princess  Spice  Tarnie  Bill  Bramble  Coal  Dunstan  Franky  Goesover  Heidi  Jet  Joseph  Khan  Kirk  Mary  Pooky  Sundance | 2020  2020  2020  2020  2020  2020  2020  2020  2020  2020  2020  2020  2021  2021  2021  2021  2021  2021  2021  2021  2021  2021  2021  2021  2021  2021 | M  M  F  M  M  F  M  F  M  F  F  F  M  M  M  M  M  M  F  M  M  M  M  F  F  M | Anglo Nubian  Saanen  Pygmy  Pygmy  Anglo Nubian  Toggenburg  Anglo Nubian  Swiss-Alpine Cross  Pygmy  Anglo Nubian  Anglo Nubian  Golden Guernsey  Toggenburg  Pygmy  Mixed Breed  Pygmy  Saanen Cross  Saanen  Toggenburg  Pygmy  Pygmy  Pygmy  Pygmy  British Alpine  Pygmy  Pygmy | 9  15  11  7  4  8  4  10  9  4  7-8  16  ≈16  Unknown  ≈8  9  5  Unknown  11  9  7+  9+  9+  16+  9  13 | 9  7  9  5  3  3  3  9  0.75  3  3  4  4  ≈3  2  7  3  1  9  4  6  4  4  4  5  7 | C, C, I, I  C, I, I  C, I, C  C, I, I, C  I, I, C, C  C, C, I, I  C, I, C, I  I, C, I  I, I, C  I, C, I  C, I  I, C, I, C  I, C  I, I, C, C  C, I, C  I, C, C, I  I, C, I, C  C, I, C  I, C, C  I, C  C, I, C  C, C, I, I  C, I, C  C, I, C  C, C, I  I, C, C | Y  Y  N  N  Y  N  Y  Y  N  Y  N  Y  N  Y  N  N  Y  Y  Y  Y  N  N  Y  Y  N  Y |

**
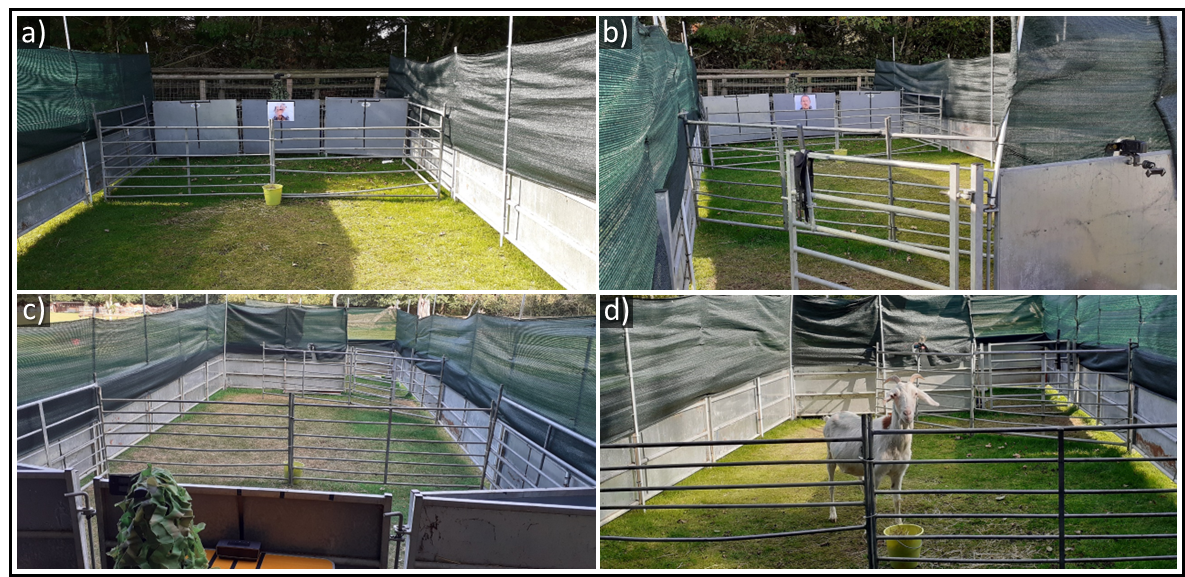
**

**Figure S1:** (a) A photograph showing the view of the front of the arena in 2020. The target bucket used to manoeuvre goats into a suitable position for stimuli presentation was placed directly in front of the photograph and speaker behind the front separator. (b) A view of the front of the arena in 2020, including the holding pen and video camera positions. (c) A view of the back of the arena, including the video camera (hidden under camouflage netting) and speaker position. (d) This photograph shows a subject in an ideal position for stimuli presentation. Image credits: Marianne Mason.

**Table S2:**

**Predictors of time taken for goats to look at the facial photograph following playbacks of a familiar person’s voice (lognormal GLMM).**

Results shown are from a basic model including only the effect of congruency and playback number. Results concerning the primary effect of interest, congruency is shown in bold.

| **Explanatory Variable** | β | S.E. | z-value | *p*-value |
| --- | --- | --- | --- | --- |
| Intercept  **Congruency (I)^a^**  Playback No. (2)^b^ | 0.558  **0.003**  -0.031 | 0.123  **0.097**  0.099 | **0.030**  -0.316 | **0.976**  0.752 |

Key: I = Incongruent; Reference Categories: a = Congruent; b = Playback Number 1

**Table S3**

**Predictors of how long goats looked at the photograph following voice playbacks (Tweedie GLMM).**

Results shown are from a basic model including only the effect of congruency and playback number. Results concerning the primary effect of interest, congruency are shown in bold.

| **Explanatory Variable** | β | S.E. | z-value | *p*-value |
| --- | --- | --- | --- | --- |
| Intercept  **Congruency (I)^a^**  Playback No. (2)^b^ | 1.100  **0.094**  -0.18402 | 0.138  **0.145**  0.13458 | **0.649**  -1.367 | **0.516**  0.172 |

Key: I = Incongruent; Reference Categories: a = Congruent; b = Playback Number 1

**Table S4:**

**Predictors of goat heart rate and HRV, relative to baseline values measured before the playback sequence was initiated) in the response period following presentation of a familiar person’s voice (LMM).**

Results shown are from a basic model including only the effect of the congruency and playback number interaction. Results concerning the primary effect of interest, the congruency x playback number interaction is shown in bold.

| **Explanatory Variable** | **Heart Rate (BPM)** | | | |  | **Heart Rate Variability (RMSSD)** | | | |
| --- | --- | --- | --- | --- | --- | --- | --- | --- | --- |
|  | β | S.E. | t-value | *p*-value |  | β | S.E. | t-value | *p*-value |
| Intercept  Congruency (I)^a^  Playback No. (2)^b^  **Congruency (I)^a^ x Playback No. (2)^b^** | -3.730  2.354  -1.016  **-6.242** | 2.631  3.460 1.779  **2.405** | 0.680  -0.571  **-2.596** | 0.500  0.572  **0.014 *** |  | 6.140  -6.552  -6.635  **10.276** | 2.684 3.439  3.607**4.879** | -1.905  -1.840  **2.106** | 0.061  0.071  **0.039 *** |

Key: I = Incongruent; Reference Categories: a = Congruent; b = Playback Number 1

**Table S5:**

Deviations in goat heart rate (BPM) from baseline values relative to the effect of congruency between human visual and vocal cues, in combination with playback number (results of post hoc tests for congruency x playback number interaction). Significant pairwise comparisons are shown in bold.

| **Explanatory Variable** | β | S.E. | t-ratio | *p*-value |
| --- | --- | --- | --- | --- |
| C1 - C2  **I1 - I2**  C1 - I1  C1 - I2  I1 - C2  C2 - I2 | 0.756  **7.198**  -1.253  5.945  2.009  5.189 | 1.850  **1.641**  4.261  4.232  4.241  4.216 | 0.409  **4.385**  -0.294  1.405  0.474  1.231 | 0.977  **0.0006*****  0.991  0.505  0.964  0.612 |

Key: C1= Congruent condition, Playback 1; C2= Congruent Condition, Playback 2; I1= Incongruent Condition, Playback 1; I2= Incongruent Condition, Playback 2. ****p* < 0.001

**Table S6:**

Changes in goat HRV (RMSSD) from baseline relative to the effect of congruency between the human face and voice playback presented, in combination with playback number (results of post hoc tests for congruency x playback number interaction).

| **Explanatory Variable** | β | S.E. | t-ratio | *p*-value |
| --- | --- | --- | --- | --- |
| C1 - C2  I1 - I2  C1 - I1  C1 - I2  I1 – C2  C2 – I2 | 6.353  -4.252  6.421  2.169  -0.068  -4.184 | 3.615  3.199  4.102  3.941  4.169  4.029 | 1.758  -1.329  1.565  0.550  -0.016  -1.039 | 0.308  0.552  0.408  0.946  1.000  0.728 |

Key: C1= Congruent condition, Playback 1; C2= Congruent Condition, Playback 2; I1= Incongruent Condition, Playback 1; I2= Incongruent Condition, Playback 2.
